# Foundations and Applications of Logotherapy to Improve Mental Health of Immigrant Populations in the Third Millennium

**DOI:** 10.3389/fpsyt.2020.00451

**Published:** 2020-06-03

**Authors:** Shirin Rahgozar, Lydia Giménez-Llort

**Affiliations:** ^1^Department of Psychiatry and Forensic Medicine, School of Medicine, Universitat Autònoma de Barcelona, Barcelona, Spain; ^2^Institut de Neurociències, Universitat Autònoma de Barcelona, Barcelona, Spain

**Keywords:** logotheraphy, mental health, therapy, immigrant, asylum, refugees

## Introduction

**Figure 1:**
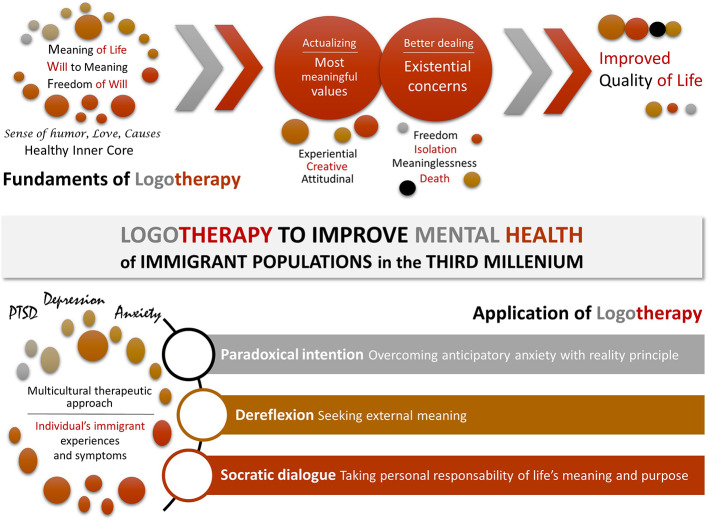
Graphical representation of the introduction.

Although migration is a natural phenomenon, a number of special conditions of this third millennium result in a rising number of populations exposed, worldwide, to the impact of risk factors for mental health associated to immigration and asylum. In this scenario, the vulnerability to distress and mental health problems such as depression, anxiety disorders and PTSD is increased in these immigrant populations due to the severity of traumatic experiences while struggling in their attempts to reach their destinations but also a poor capacity of immigrant/host to handle the situation once there. The risk for psychological and mental health problems worse with time, failure of expectancies, lack of knowledge of resources or lack of support, among other difficulties. Here, we analyzed the foundations and applications of logotherapy on immigrant mental health problems, showing the benefits that ‘finding meaning and purpose in live’ can have in these populations. Thus, among the interventions that urge to reduce depression and anxiety symptoms among older adult immigrants, we propose three logotherapy techniques, namely, paradoxical intention, dereflection and Socratic dialogue. After development and adaptation to the specific scenarios (populations/destinations) these logotherapy techniques can have a huge potential to become effective therapeutic strategies improving the mental health of the current immigrant populations facing misfortune in the Third Millennium (**Figure 1**).

## Mental Health Problems Among Immigrants in the Third Millenium

Migration has been a natural phenomenon throughout the history of humanity resulting in a melting pot of civilizations, cultures and races. However, in the words of the UN Refugee Agency (1), the world is now witnessing the highest levels of displacement on record. The Global Migration Data Portal notes that in 2019 (2) the number of international people residing in a country other than their own reached 272 million (3.5% of the global population), 51 million more than in 2010. They also highlight an important issue: although many of these individuals migrate out of choice, many others migrate out of necessity. Thus, at the end of 2018, the number of globally forcibly displaced people topped an unprecedent 70.8 million, including almost 26 million refugees, 3.5 million asylum seekers, and over 41 million internally displaced persons.

Immigrants face several life-threatening situations such as war, violence and persecution as well as losses during their journey, which hugely compromise their quality of life and mental health (3). These challenges are augmented by post-migratory stressors, such as tense asylum procedures, poverty, isolation, marginalization, fragmented social networks, discrimination and racism, acculturation, language barriers, poor access to healthcare and consequent compromised therapeutic approaches, among others (4–8). Coping with these challenges sometimes results in mental health problems and illnesses, particularly among low socio-economic backgrounds and those who are more vulnerable such as women, children, the elderly (9, 10). Immigrants experience injustices, human-instigated incidents of trauma, and subsequently respond with anger or anger-related responses. A study on Holocaust survivors and American war veterans showed that anger responses were crucial to their experiences (11). This trend was also shown in post-migration contexts, with anger responses developing independently of anxiety and depressive behavior. In these studies, morality was found to act both as protective and survival factors in the victims’ anger behavior.

It’s known that people with post-traumatic stress disorder (PTSD) - the most prevalent mental health problem in these immigrants - are also more likely to experience other mental problems, including anxiety, depression, and substance abuse disorder (12). Despite the well-researched effects of PTSD, studies indicated that the contemporary PTSD models fail to capture the complexity, human variation, and international characters of the migration-related traumas (13–15). As such, the available interventions suggest the uncontrollability of these problems, thus threatening the overall quality of life of the patients and their communities. Nevertheless, immigrant populations remain highly under-diagnosed compared to the rest of the population, thus suffering from low treatment levels (16). Moreover, mental health interventions used are limited in several ways when dealing with the immigrant population (17). Limitations include lack of sufficient evidence on specific mental problems immigrant populations suffer from, therapeutic models developed from studies conducted in Europe and North America, assumptions that specific mental health therapies can be applied across all populations and non-consideration of cultural diversity in applying mental health problems (18–20). Though mental health risks among immigrants are well documented, there are still multiple gaps in understanding barriers to access and the use of mental health services among various groups of immigrants (21).

In order to develop an effective intervention model it is important to evaluate the patients’ entire pre-migration, migration, and post-migration stages as they lead to different levels of risks to mental illnesses (22). Interventions should be based on hope and building confidence and optimism (23). They should also leverage the patients’ spirituality, religion, and other cultural factors (24). Logotherapy is thus suggested to have the potential to assist patients in finding new meaning to life and cope effectively with the past and present stressful experiences (25).

## Foundations of Logotherapy

Logotherapy is a meaning-based, value-centered psychiatric therapy developed from the works of Viktor Frankl in the early 2000s. He based logotherapy on the principle that the main motivational force of human beings is to find purpose or meaning in life. Frankl held that no other psychotherapy existed except the theory of mankind. Though he agreed that a man would never be free from all conditions, his experience of life inside the Nazi concentration camps made him believe that man has the capability of resisting and braving the most terrible conditions (26). Therefore, the primary premise of logotherapy is to help patients respond to suffering in a more meaningful way (27–29). This approach is based on the argument that mental health patients who found meaning can realize their potential and cope with their struggles in a more effective manner. Logotherapy assumes that every human person has a healthy inner core consisting of unique human and adaptive attributes such as sense of humor, love, and capacity to undertake different causes in life. The primary elements of logotherapy include the meaning of life, will to meaning as well as freedom of will (30). Based on these elements, the intervention aims at actualizing the most meaningful values to the patient meaning thereby that there is a purpose for human life (31). Will to meaning implies that the person should be able to access their unique human attributes and should be motivated to make personal sense of life, actions, and actual approach to life. Freedom of will refers to the recognition that the patients have choices and imperative to take responsibility for their choices (32).

A person discovers meaning when she/he actualizes personally meaningful values. For this purpose, the person must be committed to defining what they value i.e., experiential value, creative value, or attitudinal value (33). Experiential values refer to experiences, such as love relationships, which the person picks from their environment. Creative values include skills and behaviors associated with work, hobbies, and creative endeavors and usually earn tangible outcomes for the person (34). Attitudinal values are expressed in the manner one approaches life. Logotherapy focuses on the person’s ability to make responsible decisions that are adaptive and consistent with their meaningful values (35). In this regard, logotherapy insists that despite the person’s circumstances, life still has meaning. Finding meaning in one’s life should, therefore, be their primary motivation. Furthermore, all human persons are free to find meaning to whatever they are experiencing in their lives (36). A person’s psychological wellbeing would be compromised if their search for meaning is blocked or hindered (37). It is interesting to note that, currently, the transitions from biomedical to emergent recovery-oriented practices in mental health care are also person-centered approaches based on the premises that it is possible to adapt to a mental health condition and that personally centered meaningful goals will contribute to the recovery (38–40).

Most importantly, logotherapy approaches help the person to deal with their four existential concerns of freedom, isolation, meaninglessness, and death. According to Frankl (15, 41), freedom is an existential concern as every person should be able to determine who and what they are, while isolation is the reality that there is a gap in existence between the person and others in the society. Addressing these existential concerns requires effective psychological or psychiatric interventions intended to empower people to find meaning in work, suffering, and relationships (42). Immigrants are free to pursue the meaning from their experiences and how they have responded to the experiences (43). Through this process, an immigrant having a mental health problem can find meaning to life that helps him/her to readjust their attitudes and perceptions of potentially adverse conditions in their life journeys. After finding meaning to these difficult situations, the person will emerge, stronger, safer and happier, thus leading a resilient improved quality of life (44).

## Application of Logotherapy to Treat Immigrants in the Third Millennium

Logotherapy is developed from a multicultural lens, thus making it potentially more effective when dealing with mental health problems affecting populations of immigrants from diverse backgrounds and places all over the world (45). It leverages on the specific person’s beliefs and spirituality in finding meaning to their lives and enhancing their psychological wellbeing (46). Though logotherapy was initially designed to build faith and relationship with God, Crumbaugh and Henrion (47) found that it can be effectively applied to deal with different mental health problems, especially PTSD among immigrants who have endured difficult and stressful conditions, such as war and persecutions (48). Logotherapy follows a philosophy of phenomenological reality. Based on it, logotherapy suggests that the patient is an active participant in their treatment as he/she is an expert in their perceptions of their situations. Logotherapy not only focuses on building the relationship with the higher power but also recognizes that there is a vast variety of cultures around the world, and members of these cultures face distinct existential dilemmas of isolation and meaninglessness. Thus, Asian cultures dealt with existential meaninglessness and isolation as they work their reality towards Nirvana and ultimate transcendence (49).

Generally, logotherapy interventions are based on three primary techniques: paradoxical intention, dereflection, and Socratic dialogue. Paradoxical intention is an attempt to help clients face the situations they are most afraid of (50). This technique works by establishing the anticipatory anxiety that the immigrant is suffering from that is making it hard for them to move forward. Here, the immigrant is guided to overcome anxiety without the use of medications. On the other hand, dereflection is developed from the idea that when a person is suffering from mental health problems, such as anxiety, they are more likely to become hyper-reflective, thus focusing more on themselves and their perceptions (51). The dereflection technique helps to deflect internalization that manifests in perpetual self-examination and assist in seeking external meaning to the experiences and behaviors (52). Lastly, the socratic dialogue technique is an interview-based therapy where questions are asked in a manner that guides the client to take personal responsibility for their life’s meaning and purpose (53). Questions asked here are designed to assist the patient in finding meaning to traumatic experiences (54). Usually, this technique involves a counsellor who helps in midwifing the knowledge and capability of the patients into their consciousness (55).

To tackle mental health problems that immigrants are suffering, there is need to recognize the diversity of the immigrants’ populations, extent of mental health problems, and diagnosis and treatment limitations, among others before drawing intervention plan (56–58). In mental health intervention, it is important to consider all the three techniques of logotherapy as people possess different belief systems and will require different approaches to their problems (59). Thus, in the Connecticut in-patient veteran’s use, mandatory community service to develop resilience failed in some patients as they increase anticipatory anxiety negatively affecting the road to wellness. (60). In this case, dereflection can be augmented with dialogues to help these patients acquire coping mechanisms that they can apply whenever they experience trauma (61).

In many cases, immigrants are traumatized by the experiences of terrorists attacking travelling buses (62). In such cases, paradoxical intention assists the patient to focus on the other times where he/she made it home safely without any incidences. The technique is based on a reality principle relying on patients understanding the reality of their expectations (63). This process helps the client in readjusting the expectations when going out next time. It is essential to note that many immigrants of violence and conflicts often face the problem of perpetual self-observation to rationalize their trauma regardless of where they come from. In this case, dereflection procedures are applied to help in minimizing their vulnerability to thinking of the stressful events that may increase the chance of depressive disorders (64).

In conclusion, the effectiveness of logotherapy is demonstrated in helping patients to find meaning and purpose in their experiences and lives, and this can be applied to various mental health problems that immigrants face. Three techniques, namely paradoxical intention, dereflection, and Socratic dialogue, empower immigrants to accept their responsibility to live a meaningful life. Nonetheless, studies of logotherapy on immigrant populations are still scarce. We, therefore, suggest future studies should focus more on logotherapy applications and the development of effective therapy for diverse groups of immigrants.

## Author Contributions

Concept and review design: LG-L. Concept and review development: SR. Scientific discussions: LG-L and SR. Graphical Abstract: LG-L. Drafting manuscript: SR. Critical revision of manuscript: SR and LG-L. Approving final version of manuscript: SR and LG-L.

## Funding

Financial support provided by It’s all for L.O.V.E. projects Charity Organization, 4LOVEprojects/KF/050320.

## Conflict of Interest

The authors declare that the research was conducted in the absence of any commercial or financial relationships that could be construed as a potential conflict of interest.

## References

[1] UNHCR (2020). The United Nations Refugee Agency. https://www.unhcr.org/ (2020).

[2] Migration Data Portal http://migrationdataportal.org International Organization for Migration (IOM) (2020).

[3] Chang C Ting C Migratory Loss and Depression among Adult Immigrants of Chinese Descent. [dissertation/master"s thesis]. [Chicago (IL)]: Loyola University Chicago (2015).

[4] Hovy JD Acculturative stress, social sufficiency, and suicidal ideation in Mexican immigrants. Cult Diversity Etic Minor Psychol (2000) 6:134–51. 10.1037/1099-9809.6.2.134 10910528

[5] Connolly CM Clinical issues with same-sex couples: A review of the literature. J Couple Relationship Ther (2004) 3:3–12. 10.1300/J398v03n02_02

[6] Amri S Bemak F Mental Health Help-Seeking Behaviors of Muslim Immigrants in the United States: Overcoming Social Stigma and Cultural Mistrust. JMMH (2013) 7 (1). 10.3998/jmmh.10381607.0007.104

[7] Haynes C Identity, transcendence and the true self: Insights from psychology and contemplative spirituality. HTS Teolog Stud Theolog Stud (2016) 72:1–9. 10.4102/hts.v72i4.3455

[8] Grace F Unconditional Love in the Face of Hatred: Applications of a Timeless Teaching. Int J Philosophy Theol (2017) 5:5750–69. 10.15640/ijpt.v5n2a1

[9] Guttmann D Finding meaning in life, at midlife and beyond: Wisdom and spirit from logotherapy. Westport, CT: Praeger (2008).

[10] Brunelli C Bianchi E Murru L Monformoso P Bosisio M Gangeri L Italian validation of the Purpose in Life (PIL) test and the Seeking of Noetic Goals (SONG) Test in a population of cancer patients. Support Care Cancer (2012) 20:2775–83. 10.1007/s00520-012-1399-6 22350595

[11] Stegger MF Oishi L Is a life without meaning satisfying? J Gerontol Ser B (2004) 54:125–35.

[12] Schluckebier ME (2013). Dreams worth pursuing: how college students develop and articulate their purpose in life. University of Iowa. 10.17077/etd.q5gontba

[13] Jahanpour Z Sareghin SA Hosseini FS Tekiyee A The study of group logotherapy effectiveness on self-esteem, happiness and social sufficiency in Tehranian girl teenagers. J Med Sci (2014) 7:477–89.

[14] Graber AV Viktor Frankl"s logotherapy: Method of choice in ecumenical pastoral psychology. Lima, OH: Wyndham Hall (2003).

[15] Frankl V Man's Search for Meaning. An Introduction to Logotherapy. Boston, MA: Beacon Press (2006).

[16] Nassif C Schulenberg S Hutzell RR Rogina J Clinical Supervision and Logotherapy: Discovering Meaning in the Supervisory Relationship. J Contemp Psychother (2009) 40:21–9. 10.1007/s10879-009-9111-y

[17] Mosalanejad L Koolee AK Looking at Infertility Treatment through The Lens of The Meaning of Life: The Effect of Group Logotherapy on Psychological Distress in Infertile Women. Int J Fertil Steril (2013) 6:224–31. PMC3850317 24520444

[18] Sunhee C Kunsook S Bernstein Soonhee R Daniel C Chen Logo-Autobiography and Its Effectiveness on Depressed Korean Immigrant Women. J Transcult Nurs (2013) 24:33–42. 10.1177/1043659612452005 22802301

[19] Fabry DDS Sheikh A Selman M Logotherapy can enrich cognitive behavioral therapy practice. Int Forum Logother (2007) 30:100–6.

[20] Zotova N Religion and Mental Health among Central Asian Muslim Immigrants in Chicago Metropolitan Area. Migration Lett (2018) 15:361–76. 10.33182/ml.v15i3.358

[21] Kwong K Chung H Cheal K Chou JC Chen T Depression care management for Chinese Americans in primary care: a feasibility pilot study. Community Ment Health J (2011) 49:157–65. 10.1007/s10597-011-9459-9 22015960

[22] Toumisto MT Roche JE Beyond PTSD and Fear-Based Conditioning: Anger-Related Responses Following Experiences of Forced Migration—A Systematic Review. Front Psychol (2018). 9:2592 10.3389/fpsyg.2018.02592 30619002 PMC6306035

[23] Anagnostopoulos D Communications of the European Society for Child and Adolescent Psychiatry. ECAP (2016) 25:119–22. 10.1007/s00787-019-01315-7 30972582

[24] Savolaine J Granello PF The function of meaning and purpose for individual wellness. J Humanistic Counsel Educ Dev (2002) 41:178–89. 10.1002/j.2164-490X.2002.tb00141.x

[25] Karadems EC Self-efficacy, social support and well-being the mediating role of optimism. J Pers Individ Dif (2006) 40:1281–90. 10.1016/j.paid.2005.10.019

[26] Marshall M Marshall E Logotherapy revisited, review of the tents of Viktor E. Frankl's Logotherapy. Ottawa Institute of Logotherapy (2012). 284 p.

[27] Addad EM Himi H Logotherapy –Theoretical Aspects and Field Studies in Israel. IJHSS (2015), 5.

[28] Schulenberg SE Henrion RP Logotherapy past, present, and future: A conversation with James C. Crumbaugh. Int Forum Logother (2005) 28:65–71. 10.1037/a0014331

[29] Robatmili S Sohrabi F Shahrak MA Talepasand S Nokani M Hasani M The Effect of Group Logotherapy on Meaning in Life and Depression Levels of Iranian Students. Int J Advancement Counsel (2015) 37:54–62. 10.1007/s10447-014-9225-0 PMC4355442 25774068

[30] Chetan J Jacob M Marszalek LaVerne A Berkel Adam B An Empirical Investigation of Viktor Frankl’s Logotherapeutic Model. J Humanistic Psychol (2014) 54:227–53. 10.1177/0022167813504036

[31] Schulenberg S Hutzell RR Nassif C Rogina J Logotherapy for clinical practice. Psychother Theory Res Pract (2008) 45:447–63. 10.1037/a0014331 22122533

[32] Schulenberg SE Psychotherapy and movies: On using films in clinical practice. J Contemp Psychother (2003) 33:35–48. 10.1023/A:1021403726961

[33] Hutchinson GT Chapman BP Logo therapy -enhance REBT: an integration of discovery and reason. J Contemp Psychother (2005) 35:145–50. 10.1007/s10879-005-2696-x

[34] Melton AMA Schulenberg SE On the measurement of meaning: Logotherapy’s empirical contributions to Humanistic psychology. Humanistic Psychol (2008) 36:1–14. 10.1080/08873260701828870

[35] Moreno MA Jelenchick LA Egan KG Cox E Young H Gannon E Feeling bad on Facebook: depression disclosures by college students on a social networking site. Depression and Anxiety. Wisconsin, USA: School of Medicine and Public Health, University of Wisconsin, Madison (2011) 28:447–455. 10.1002/da.20805 21400639 PMC3110617

[36] Antoniades J Mazza D Brijnath B Efficacy of depression treatments for immigrant patients: results from a systematic review. BMCP (2014) 14:176. 10.1186/1471-244X-14-176 PMC4084503 24930429

[37] Devoe D Viktor Frankl’s Logotherapy: The Search for Purpose and Meaning. Inquiries J Pulse (2012) 4 Available at: http://www.inquiriesjournal.com/a?id=660 (Accessed March 3, 2020).

[38] Leamy M Bird V Le Boutillier C Williams J Slade M Conceptual framework for personal recovery in mental health: systematic review and narrative synthesis. Br J Psychiatry (2011) 199(6):445–52. 10.1192/bjp.bp.110.083733 22130746

[39] Strand M Gammon D Ruland CM Transitions from biomedical to recovery-oriented practices in mental health: a scoping review to explore the role of Internet-based interventions. BMC Health Serv Res (2017) 17(1):257. 10.1186/s12913-017-2176-5 28388907 PMC5385090

[40] Winsper C Crawford-Docherty A Weich S Fenton SJ Singh SP How do recovery-oriented interventions contribute to personal mental health recovery? A systematic review and logic model. Clin Psychol Rev (2020) 76:101815. 10.1016/j.cpr.2020.101815 32062302

[41] Frankl VE On the theory and therapy of mental disorders: An introduction to logotherapy and existential analysis. DuBois JM, editor. New York: Brunner-Routledge (2004).

[42] Haditabar H Smaeeli Far N Amani Z Effectiveness of Logotherapy concepts training in increasing the quality of life among students. Int J Psychol Behav Res (2013) 2:223–30.

[43] Spore AP Meaning-centered love: Foundation of meaningful marriage. Int Forum Logother (2008) 31:96–102.

[44] Costanza A Prelati M Pompili M The Meaning in Life in Suicidal Patients: The Presence and the Search for Constructs. A Systematic Review. Medicina (2019) 55. 10.3390/medicina55080465 PMC6723920 31405240

[45] Aguinaldo JL De Guzman R The effectiveness of logo-bibliotherapy on the depression of selected Filipinos suffering from Myasthenia Gravis. AJNAS (2014) 3:31–9.

[46] Rahiminezhad A Kazemi Z Farahani H Aghamohamadi S Purpose in life and identity dimensions as predictors of maladaptive psychological aspects: a path analysis study. Soc Behav Sci (2011) 30:1009–13. 10.1016/j.sbspro.2011.10.196

[47] Crumbaugh JC Henrion R The power of meaningful intimacy: Key to successful relationships. Philadelphia, PA: Xlibris (2004).

[48] Yalom ID When Nietzsche wept, a novel of obsession. New York: Harper Perennial (2005).

[49] Florez IA (2017). On the Relationship between Meaning and Prejudice: Examining Self- Transcendence and Value-Behavior Consistency in a Sample of College Students University of Mississippi.

[50] Pytell T Transcending the Angel Beast: Viktor Frankl and Humanistic Psychology. Psychoanalyt Psychol (2006) 23:490–503. 10.1037/0736-9735.23.3.490

[51] Feldman DB Snyder CR Hope and the meaningful life: Theoretical and empirical associations between goal-directed thinking and life meaning. J Soc Clin Psychol (2005) 24:401–21. 10.1521/jscp.24.3.401.65616

[52] Cho S Bernstein KS Roh S Logo-Autobiography and Its Effectiveness on Depressed Korean Immigrant Women. J Transcult Nurs (2013) 24:33–42. 10.1177/1043659612452005 22802301

[53] Julom AM De Guzman R The Effectiveness of Logotherapy Program in Alleviating the Sense of Meaninglessness of Paralyzed In-patients. Int J Psychol psychol Ther (2013) 13:357–71.

[54] Segal DL Coolidge FL O’Riley A Heinz BA Structured and semi-structured interviews. In: Hersen M, editor. Clinician"s handbook of adult behavioral assessment. Boston, MA: Elsevier Academic Press (2006). p. 121–44.

[55] Morgan JH Late-Life depression and the counseling agenda: exploring geriatric logotherapy as a treatment modality. Int J psychol Res (2012) 5:99–105.

[56] Duba JD Watts RE Therapy with religious couples. J Clin Psychol: Session (2009) 62:210–23. 10.1002/jclp.20567 19123233

[57] Mendez M A life with meaning: Guide to the fundamental principles of Viktor E. Frankl’s logotherapy. Victoria, BC: Trafford (2004).

[58] Niemiec RM Wedding D Positive psychology at the movies: Using films to build virtues and character strengths. Cambridge, MA: Hogrefe & Huber (2008).

[59] Fahimeh M Fard FD Heidari H Effectiveness of Logo Therapy in Hope of Life in the Women Depression. Proc Soc Behav Sci (2014) 159:643–6. 10.1016/j.sbspro.2014.12.440

[60] Schulenberg SE Schnetzer LW Winters MR Hutzell RR Meaning-Centered Couples Therapy: Logotherapy and Intimate Relationships. J Contemp Psychother (2010) 40:95–102. 10.1007/s10879-009-9134-4

[61] Somov PG Meaning of life group: group application of logotherapy for substance use treatment. J Specialists Group Work (2007) 32:316–45. 10.1080/01933920701476664

[62] Tribe RH Sendt K Tracy DK A systematic review of psychosocial interventions for adult refugees and asylum seekers. JMH (2019) 28:662–76. 10.1080/09638237.2017.1322182 28485636

[63] Eisenberg D Golberstein E Gollust SE Help-seeking and access to mental health care in a university student population. Med Care (2007) 45:594–601. 10.1097/MLR.0b013e31803bb4c1 17571007

[64] McLafferty C “Meaning until the Last Breath”: Practical Applications of Logotherapy in the Ethical Consideration of Coma, Brain Death, and Persistent Vegetative States. In: Batthyány A, editor. Logotherapy and Existential Analysis. Logotherapy and Existential Analysis, vol. 1 . (2016). p. 365–76.

